# In Vivo Characterization of Tick-Borne Encephalitis Virus in Bank Voles (*Myodes glareolus*)

**DOI:** 10.3390/v11111069

**Published:** 2019-11-15

**Authors:** Anna Michelitsch, Birke Andrea Tews, Christine Klaus, Malena Bestehorn-Willmann, Gerhard Dobler, Martin Beer, Kerstin Wernike

**Affiliations:** 1 Institute of Diagnostic Virology, Friedrich-Loeffler-Institut, Südufer 10, 17493 Greifswald—Insel Riems, Germany; anna.michelitsch@fli.de; 2 Institute of Infectology, Friedrich-Loeffler-Institut, Südufer 10, 17493 Greifswald—Insel Riems, Germany; birke.tews@fli.de; 3 Institute for Bacterial Infections and Zoonoses, Friedrich-Loeffler-Institut, Naumburger Str. 96a, 07743 Jena, Germany; christine.klaus@fli.de; 4 Department of Parasitology, University of Hohenheim, Schloss Hohenheim 1, 70599 Stuttgart, Germany; malenabestehorn-willmann@bundeswehr.org (M.B.-W.); gerharddobler@bundeswehr.org (G.D.); 5 Bundeswehr Institute of Microbiology, German Center of Infection Research (DZIF) partner site Munich, Neuherbergstraße 11, 80937 München, Germany

**Keywords:** tick-borne encephalitis virus, bank vole, experimental infection, virus detection, reservoir host

## Abstract

Tick-borne encephalitis is the most important tick-transmitted zoonotic virus infection in Eurasia, causing severe neurological symptoms in humans. The causative agent, the tick-borne encephalitis virus (TBEV), circulates between ticks and a variety of mammalian hosts. To study the interaction between TBEV and one of its suspected reservoir hosts, bank voles of the Western evolutionary lineage were inoculated subcutaneously with either one of eight TBEV strains or the related attenuated Langat virus, and were euthanized after 28 days. In addition, a subset of four strains was characterized in bank voles of the Carpathian linage. Six bank voles were inoculated per strain, and were housed together in groups of three with one uninfected in-contact animal each. Generally, most bank voles did not show any clinical signs over the course of infection. However, one infected bank vole died and three had to be euthanized prematurely, all of which had been inoculated with the identical TBEV strain (Battaune 17-H9, isolated in 2017 in Germany from a bank vole). All inoculated animals seroconverted, while none of the in-contact animals did. Viral RNA was detected via real-time RT-PCR in the whole blood samples of 31 out of 74 inoculated and surviving bank voles. The corresponding serum sample remained PCR-negative in nearly all cases (29/31). In addition, brain and/or spine samples tested positive in 11 cases, mostly correlating with a positive whole blood sample. Our findings suggest a good adaption of TBEV to bank voles, combining in most cases a low virulence phenotype with detectable virus replication and hinting at a reservoir host function of bank voles for TBEV.

## 1. Introduction

Tick-borne encephalitis (TBE) is a severe neurological disease that can lead to long-lasting sequelae, burdening the affected patient for years [1]. Even though effective vaccination is possible, there are still 2000 to 4000 cases reported yearly in the European Union alone, where TBE has been a notifiable disease since 2012 [2]. Worldwide, there are more than 10,000 cases reported each year; the highest percentage of cases diagnosed in Russia [3]. Since TBE surveillance in the northern parts of Asia is not yet regularly conducted except for in Russia, the actual number of cases may be even higher [4,5]. Overall, case numbers tend to fluctuate over time [6,7], since transmission rates to humans are dependent on multiple factors [8,9,10]. The causative agent, the tick-borne encephalitis virus (TBEV), is a member of the family Flaviviridae, in which it belongs to the tick-transmitted complex alongside with louping-ill virus (LIV), Langat virus (LGTV), Kyasanur Forest disease virus (KFDV), and Powassan virus (POWV), and a number of other viruses [11]. TBEV is divided into at least five subtypes: the European subtype (TBEV-Eu), the Siberian subtype (TBEV-Sib), the far-eastern subtype (TBEV-Fe), and the recently identified Himalayan and Baikalian subtypes [12,13,14]. Among factors such as the infectious dose, age, genotype, and health status of the patient [15], the subtype can influence the severity of disease in humans [6,16,17]. Immune response to TBEV infection may also play a role in disease severity and has been reviewed by Ruzek et al. [15]. Hard-bodied ticks are the central point of the transmission cycle of TBEV [6,18,19]. They spread the virus among a variety of animal species [20,21,22,23,24] and represent a virus reservoir, as they are able to retain the virus during their different life stages through trans-stadial and trans-ovarial transmission [25]. Nonetheless, an additional source of infection for naïve ticks is needed to spread the virus in the tick population and assure sufficient circulation in endemic regions [26]. This source of infection is often presumed to be a vertebrate reservoir. According to the WHO, a reservoir host is a mammalian host that ideally becomes infected without showing signs of disease and remains viremic for a long time, with titers high enough to infect a naïve vector [27]. However, there is no unified definition of the term reservoir host [28] and, therefore, there are no clear criteria a reservoir host has to fulfil [29]. In this paper, the term “reservoir host” is therefore used merely as a term to define the possibility of a host to become a relevant source of infection for an arthropod vector through the development of long-lasting viremia. The suspected vertebrate reservoir hosts for TBEV are small mammalians living on the ground of the deciduous and mixed forest ecosystems where ticks are found in abundance [30]. Alongside a process called co-feeding, where infected ticks pass the virus directly to naïve ticks through a shared feeding pool while being attached to the same animal in close proximity [31], the classical route of infection is via consumption of a blood meal from a viremic animal [32]. However, the importance of this direct transmission of TBEV from a viremic animal to a naïve tick has been questioned [33], mostly based on the fact that there are hardly any studies available on the interaction between TBEV and its putative natural hosts. Existing studies describe a viremia of three to nine days and a possible persistent infection of the brain of various small mammalian species [34,35,36,37,38]. A more recent study described a potentially longer viremia, especially after an infection with a TBEV-Fe strain in bank voles [39]. 

The present study set its focus on the situation in Europe, where *Ixodes ricinus* ticks are the main vectors and bank voles (*Myodes glareolus*) are suspected to be one of the main vertebrate reservoir hosts [40]. Bank voles are among the most frequently trapped small mammals in various European TBE monitoring studies. They are used as sentinels for TBEV circulation since both antibodies and viral RNA in considerable amounts have been found in organ samples of caught animals from known endemic regions [41]. The bank vole population is divided into different evolutionary lineages based on mitochondrial DNA (mtDNA) sequencing. These lineages originated due to the post-glacial re-colonization of Europe from bank vole colonies that survived the glaciation in different refugia. The Western lineage is found in the western parts of Europe and is separated from the Eastern lineage by the Carpathian lineage which occurs in Poland, the Slovak Republic, and Romania. In addition, Spanish, Italian, and Balkan lineages have been described [42]. 

Here, a variety of TBEV-Eu strains that were isolated from either humans, ticks, or bank voles were selected and inoculated into bank voles of the Western evolutionary linage [42]. In addition, LGTV was used, which is a lowly pathogenic virus that is similar to TBEV in its transmission cycle but not endemic in Europe [43]. Furthermore, LTGV shows antibody cross-reactivity with TBEV and was considered a vaccine candidate in early TBEV research [44]. To address the potential influence of different linages on the interaction between TBEV and the natural rodent host, as is known for, for example, Puumala orthohantavirus [45], four of these strains were also tested in bank voles of the Carpathian linage. 

The samples that were generated during this experimental infection study were further used to validate available test systems for the bank vole and to evaluate different sample matrices for their usage to detect certain parameters. 

## 2. Materials and Methods 

### 2.1. TBEV-Eu Strains

Eight TBEV-Eu strains were selected (Table 1). Seven strains were obtained from the collection of the Department of Microbiology of the German Armed Forces, Munich, Germany. The eighth strain (IZ58) and the LGTV were obtained from the virus collection of the Friedrich-Loeffler-Institut, Greifswald—Insel Riems, Germany. The selected strains were propagated on A549 cells (L 1035, Collection of Cell Lines in Veterinary Medicine (CCLV), Friedrich-Loeffler-Institut, Greifswald—Insel Riems, Germany) for one passage. 

### 2.2. Animals and Experimental Design

All seven TBEV-Eu strains as well as LGTV were inoculated into bank voles of the Western linage. Four out of these TBEV-Eu strains, namely Scharl, Battaune 17-H9, GCl 223, and IZ58, were simultaneously characterized in bank voles of the Carpathian linage.

Animal housing and all handling took place under BSL 3** conditions. Altogether, 114 outbred bank voles (*Myodes glareolus*) obtained from the in-house breeding colonies of the Friedrich-Loeffler-Institut were used. The breeding colony of the Western evolutionary lineage originated from bank voles that were provided by the Federal Environmental Agency in Berlin, Germany, and the breeding colony of the Carpathian evolutionary lineage originated from bank voles that were provided by Jagiellonian University Krakow, Poland. Serological assays are performed on a regular basis to ensure the specific pathogen-free status of both breeding colonies [50]. PCR amplification and sequencing of the partial *cytochrome b* gene was performed following a standard protocol [51]. The generated nucleotide sequences were then used in a phylogenetic analysis to confirm their affiliation to the respective evolutionary lineage [50]. Seventy-eight bank voles belonged to the Western lineage and 36 to the Carpathian lineage. The voles were kept in single-ventilated type III mouse cages under the following conditions: 22 °C; 12/12 h light cycle, approximately 60% humidity, water and rodent pellets ad libitum. To assure smooth social interaction between the voles, only female voles were selected. Admittedly, three animals turned out to be males at dissection. The animals were housed in pairs of four, ranging in age between 5 and 32 weeks at the day of infection. Three voles from each cage were inoculated subcutaneously with 100 µL virus dilution per animal, containing 10^5^ tissue culture infectious dose 50% (TCID_50_). The remaining animal acted as an in-contact animal to detect possible transmission from the infected voles. For each TBEV-Eu strain, a total of six voles were inoculated, meaning that two cage groups of three voles with one contact animal each were used per strain. Ten voles acted as environmental controls; six out of them belonged to the Western lineage and four to the Carpathian lineage. All voles were examined daily based on a clinical score system (up to three points were awarded for each changes in behavior, neurological symptoms, and loss of body weight). Weight loss of more than 20% of the original weight, paralysis of the limbs, a clinical score of seven or other clinical signs suggesting suffering were predefined as endpoint criteria. Twenty-eight days post infection (dpi), autopsy of all remaining bank voles was performed. In addition to the collection of whole blood and serum samples, 11 organs (brain, spinal cord, lung, heart, small and large intestine, liver, spleen, kidney, bladder, and uterus/testicle) were sampled. Whenever possible, samples of feces and urine were taken as well. Lastly, a lavage of the chest cavity was performed with 1 mL phosphate-buffered saline buffer (PBS). All samples were stored at −80 °C until analyzed.

The experimental design was evaluated and approved by the relevant state ethics committee (State Office for Agriculture, Food Safety and Fishery in Mecklenburg-Western Pomerania, permission number 7221.3-1.1-029/18, 28 May 2018).

### 2.3. RNA Extraction and RT-PCR

The collected organs and the feces samples were mixed with 1 mL modified Eagle’s medium (MEM) and homogenized using a TissueLyzer (Qiagen, Hilden, Germany). After centrifugation, 100 µL of the supernatant was used for RNA extraction. Urine samples were also collected in 1 mL MEM, of which 100 µL was used for extraction. Lavages were used directly (volume 100 µL). For the extraction of RNA from EDTA blood and serum, 15 µL of the sample was used. RNA extraction was performed using the King Fisher 96 Flex purification system (Thermo Scientific, Braunschweig, Germany) in combination with the NucleoMag^®^ Vet Kit (Macherey-Nagel, Düren, Germany) according to the manufacturer’s instructions. The extracts were subsequently tested for TBEV using a previously described and validated real-time RT-PCR, targeting a fragment of the 3′-untranslated region (3′UTR) of the TBEV genome [52]. The TBEV test was carried out as described; however, to control for efficient RNA extraction and amplification and thereby avoid false negative results, an internal control based on the beta-actin gene was included [53] instead of the previously described heterologous control [52].

### 2.4. Comparison of Real-Time RT-PCR to Cell-Culture Infectivity

TBEV-Eu cell culture passages that were used in the animal experiment (Table 1) were used to correlate cell-culture infectivity to real-time RT-PCR detection of viral genome. To determine the cell-culture infectivity, the viral suspension of each isolate was diluted in serum-free MEM in a 10-fold series until a dilution of 10^−8^ was reached. A459 cells suspended in MEM supplemented with 5% bovine viral diarrhea virus (BVDV)-free fetal calf serum were then added to each dilution. The described titration was performed in eight replicates and TCID_50_/mL was determined through detection of cytopathic effect (cpe).

For the detection of viral genome, the viral suspensions were diluted using serum-free MEM in a 10-fold dilution series until a 10^−12^ dilution was reached. Viral RNA was extracted and real-time PCR was performed as described above, using 100 µL of each dilution for the initial extraction. The mathematical relationship between real-time RT-PCR and the logarithmic TCID_50_/mL values of the same dilution was then modeled using simple linear regression [54]. The SigmaPlot program (Sytat Software GmbH) was used to create a graph with a single linear regression line for all TBEV-Eu strains. RT-PCR results were then used to estimate cell-culture infectivity.

### 2.5. Virus Isolation

Reisolation of viruses in cell culture was attempted on human lung carcinoma cells (A549, L 1035 CCLV, Insel Riems, Germany), cultivated in MEM supplemented with 10% BVDV-free fetal calf serum for three passages. Successful cultivation was detected through cpe on the cells and confirmed through RT-PCR. 

### 2.6. Antibody Detection

A microneutralization assay was performed according to Holzmann et al. [55], with minor modifications. Each serum sample was tested in duplicate. A set of known control sera was tested in parallel. The serum samples were first diluted in a 1:20 ratio and then titrated in 2-fold dilutions. LGTV was then added with approximately 100 TCID_50_/well, which was confirmed by performing back-titrations. A549 cells were added to the virus–serum mixture and incubated at 37 °C for seven days. Titers were evaluated via appearance of cpe and are expressed as the dilutions that caused 50% neutralization (ND_50_). Besides the collected sera, the chest cavity lavages were also tested by the microneutralization assay, following the same protocol but starting at a dilution of 1:5.

## 3. Results

### 3.1. Clinical Manifestation

None of the bank voles showed any neurological symptoms over the course of infection. A single animal of the Western lineage (inoculation with strain Battaune 17-H9) displayed signs of distress and died three days after infection, before the clinical examination score fulfilled the predefined humane endpoint criteria. 

Two voles of the Western lineage and one of the Carpathian lineage, inoculated with the same TBEV-Eu strain (Battaune 17-H9), had to be euthanized at 5, 6, or 12 dpi due to weight loss of more than 20% of the animal’s original weight. For the same reason, one contact animal, which belonged to the Western lineage, had to be euthanized 6 days after infection as well as one environmental control animal, which belonged to the Carpathian lineage, at 19 dpi; none of these animals displayed any signs of distress except for weight loss. 

### 3.2. Virus RNA Detection

**Figure 1:**
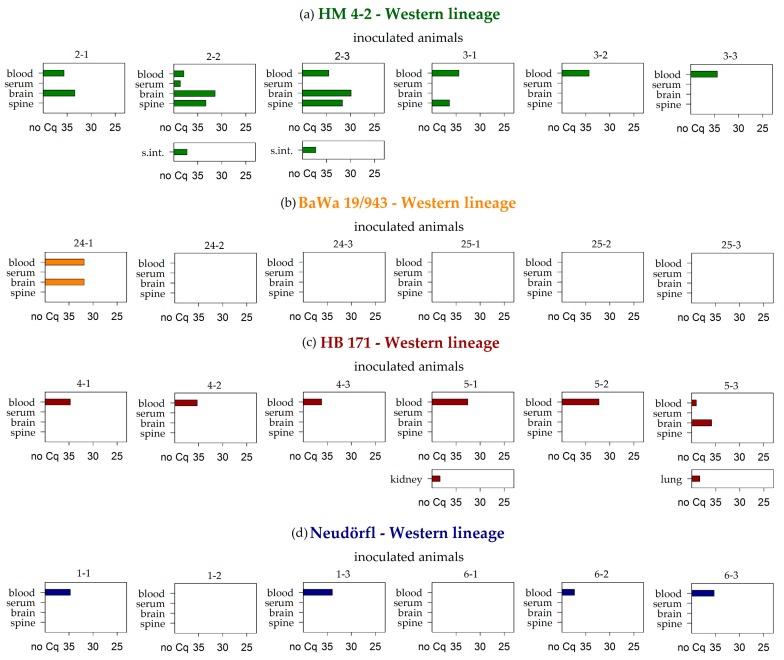
RT-PCR results of blood, serum, brain, and spine samples for the bank voles of the Western lineage that were inoculated with the (**a**) HM 4-2, (**b**) BaWa 15/943, (**c**) Neudörfl, and (**d**) HB 171 TBEV-Eu strains. Further additional positive samples are listed per animal. Measures are given in quantification cycle values (Cq). S.int.: small intestine.

**Figure 2:**
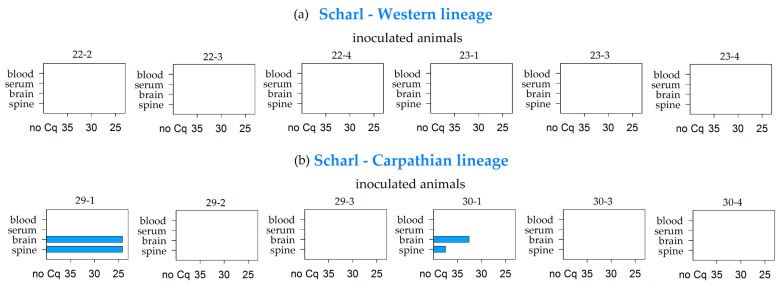
RT-PCR results of blood, serum, brain, and spine samples for the bank voles of the Western (**a**) and Carparthian (**b**) lineages that were inoculated with the Scharl TBEV-Eu strain. Measures are given in quantification cycle values (Cq).

**Figure 3:**
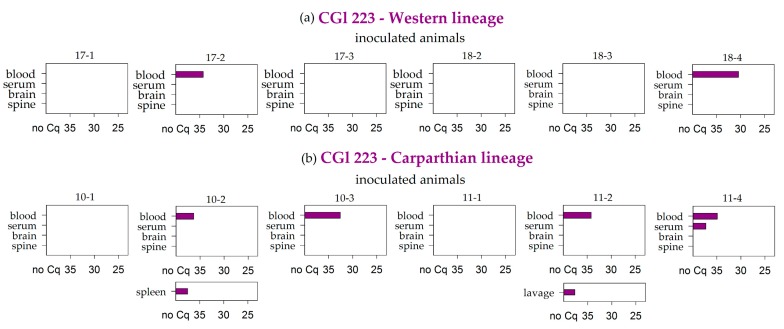
RT-PCR results of blood, serum, brain, and spine samples for the bank voles of the Western (**a**) and Carparthian (**b**) lineage that were inoculated with the CGl 223 TBEV-Eu strain. Further additional positive samples are listed per animal. Measures are given in quantification cycle values (Cq).

In general, EDTA blood represented the sample material that most frequently tested positive by real-time PCR in both vole lineages. At the end of the study (28 dpi), viral RNA was detected in whole blood samples of 31 animals out of the 74 surviving inoculated voles. The respective viremic animals had been inoculated with either the TBEV strain HB 171 (6 positive of 6 surviving inoculated animals), CGl 223 (6/12), Battaune 17/H9 (8/8), HM 4-2 (6/6), Neudörfl (4/6), or BaWa 15/943 (1/6). In contrast to this, the corresponding serum sample tested negative in most cases (29 out of 31, Table 2, Figure 1, Figure 2 and Figure 3 and Figure 5). 

Brain samples also tested positive for TBEV by RT-PCR in considerable amounts, and mostly correlated with the detection of positive whole blood samples (9 out of 31) (Table 2, Figure 1, Figure 2 and Figure 3 and 5). The spine samples tested positive in 6 out of the 31 viremic voles. In addition to these 31 animals, viral RNA was detected in 2 further voles, namely in the brain and spine sample of animals inoculated with the Scharl strain (2/12) (Table 2, Figure 2b). 

No animal inoculated with the strain IZ58, which originated from an area not endemically affected, tested positive for any examined sample. The animals inoculated with LGTV likewise tested negative in all of the analyzed samples (Table 2). 

**Figure 4:**
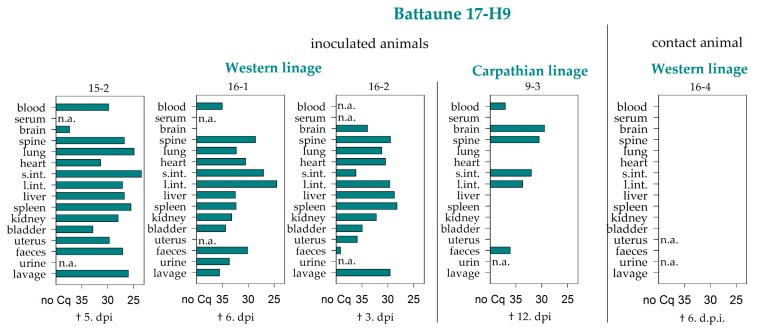
RT-PCR results of the bank voles that had to be taken out prior to the endpoint. The day of removal is given underneath the respective graph as days post infection (d.p.i). Missing samples are marked with n.a. (not available).

Interestingly, Battaune 17-H9 was the only strain that caused premature losses in both bank vole lineages. All samples of the animals that were euthanized at 3, 5, or 6 dpi tested positive in the RT-PCR, and only the brain sample from the animal that died at 3 dpi remained negative. In the animal that was prematurely euthanized at 12 dpi, viral RNA was detected in the whole blood, the brain, and the spine, as well as in the digestive tract samples (Figure 4). 

**Figure 5:**
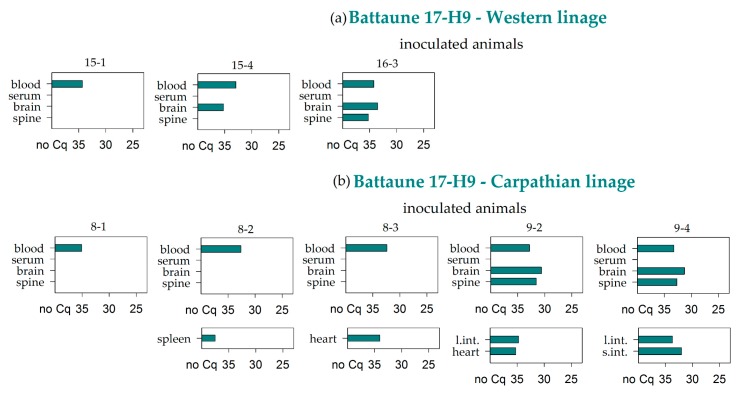
RT-PCR results of blood, serum, brain, and spine samples for the bank voles of the Western (**a**) and Carpathian (**b**) lineages that were inoculated with the Battaune TBEV-Eu strain. Further additional positive samples are listed per animal. Measures are given in quantification cycle values (Cq). S.int. and l. int.: small and large intestine.

The remaining animals that were inoculated with the Battaune 17-H9 strain also resulted positive for the whole blood samples independently of the vole evolutionary lineage. In four voles, two of each linage, the brain sample was also positive, and the corresponding spine samples tested positive in three out of these four cases (Figure 5). Every in-contact and environmental control animal tested negative by RT-PCR.

### 3.3. Comparison of Viral RNA Detection and Cell-Culture Infectivity

**Figure 6:**
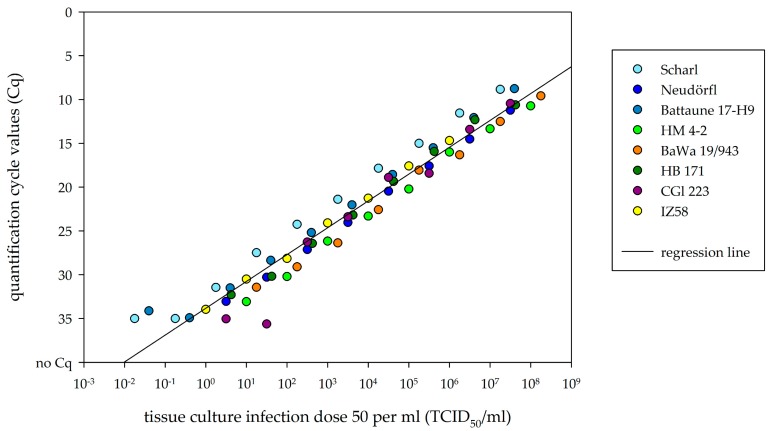
Scatter plot between logarithmic tissue culture infection dose 50 (TCID_50_)/mL and quantification cycle values (Cq) of TBEV-Eu isolates. Dots are color marked in accordance to each TBEV-Eu isolate. Regression line is drawn for the mean of all plots.

All eight TBEV-Eu strains showed a mathematical correlation between Cq value and logarithmic TCID_50_/mL value. The higher the TCID_50_/mL value, the earlier viral RNA was detected via RT-PCR, leading to lower Cq values. Scatter plot visualization showed a clustering of Cq values in accordance with TCID_50_/mL values and a single linear regression line for all TBEV-Eu isolates. Further RT-PCR managed to detect viral RNA even in dilutions with a negative TCID_50_/mL value (Figure 6). 

The RT-PCR results of the tested tissue samples showed Cq values from around 25 to 35. Estimating the infectivity on cell cultures from the single regression line leads to a TICD_50_/mL of 10^1.37^ for a Cq value of 30. The Cq values 25 and 35 led to TCID_50_/mL values of 10^2.92^ and 10^−0.18^, respectively. 

### 3.4. Virus Isolation

Virus isolation was attempted from all positive brain and spine samples, as well as from selected positive organ samples of the bank voles that died prematurely. Virus was successfully reisolated from the brain tissue of two of the bank voles that had been inoculated with the HM 4-2 strain (animals 2-2 and 2-3). From the prematurely euthanized bank voles, TBEV-Eu was reisolated from one large intestine sample (16-1), one heart sample (16-1), and one lung sample (15-2). Virus isolation from positive EDTA blood samples was attempted with the samples that had the lowest Cq values, but failed due to the pronounced cell toxicity of the samples. 

### 3.5. Comparative Antibody Detection between Sera and Lavages

In the serum samples of all surviving inoculated animals, specific neutralizing antibodies could be detected at the end of the study (28 dpi), while neither the in-contact animals nor the voles that were used as environmental controls seroconverted. Overall, all strains led to high values of neutralizing antibodies in the inoculated bank voles of the Western lineage as well as those of the Carpathian lineage (Table 3 and Table 4). 

There were no striking differences between the bank voles of the two lineages when inoculated with the same TBEV-Eu strain. For details, see Table 4.

The comparative testing of both serum samples and lavage samples showed no direct correlation. However, the values using the lavage samples were always markedly lower than the values using the corresponding serum sample. In eight lavage samples, no neutralizing antibodies were detected even though the corresponding serum sample showed a neutralization titer of at least 1:20. The neutralizing titers of all tested lavage samples did not exceeded 1:40. Only 11 out of the 78 tested lavage samples had neutralizing titers of more than 1:10. In comparison to this, 48 out of the 74 available serum samples reached neutralizing titers of 1:120 or higher (Table 3 and Table 4).

## 4. Discussion

TBEV is one of the most important tick-transmitted zoonotic pathogens [56] and can lead to severe meningoencephalitis in humans [15]. The virus is endemic in forest and grassland areas, where it is transmitted to a multitude of animal species. Among them, small mammalians are suspected to be of importance for TBEV circulation, enabling the virus to be spread among the tick population [57]. To better understand the interaction between TBEV and its putative natural hosts, the virus–host interaction was studied under experimental conditions using European strains of TBEV in Central and Carpathian European voles. 

In the present study, all TBEV-Eu strains used led to successful infection in all inoculated bank voles, as demonstrated by the detection of viral RNA and/or the presence of neutralizing antibodies. TBEV-Eu genome was found after 28 days in the whole blood samples of all bank voles that were inoculated with either HM 4-2 or HB 171/11, as well as in four out of six bank voles that were inoculated with the Neudörfl strain, suggesting a long-lasting viremia of at least up to a month. In addition, viral RNA was detected in the brain samples of numerous animals. The strain HM 4-2 was even successfully reisolated in cell culture from two positive brain samples, proving that indeed infectious virus was still present in the bank voles at 28 days post infection. For the common vole (*Microtus arvalis*), it was shown that this persistent infection in the central nervous system can potentially last for 100 days [37], which should be further explored for the bank vole. 

In comparison to TBEV-Eu, the closely related, serologically cross-reactive LTGV was used as a control. This virus also belongs to the tick-transmitted Flaviviridae complex and leads to occasional meningoencephalitis in humans, but is only endemic in Malaysia. [43]. All inoculated bank voles became infected when inoculated with LGTV, which was proven by the presence of neutralizing antibodies, but no viral RNA was detected in any samples through RT-PCR testing. This was in clear contrast to the persistent brain infection and viremia in bank voles inoculated with TBEV-Eu strains and, therefore, may indicate an efficient adaptation of the TBEV-Eu strains to the locally occurring small mammalian host.

However, the most striking result of this study was the detection of viral RNA in the whole blood sample of inoculated animals 28 days after infection, while the corresponding serum sample remained negative in most cases. This phenomenon was previously hinted at in a study conducted in the 60s [36], an experimental study of TBEV-Sib in the red vole (*Myodes rutilus*) [58], and in a trapping study that differentiated between serum and blood clots [40]. Nevertheless, this fact is often overlooked and can lead to false assumptions concerning the duration of potential viremia [39] and an underestimation of prevalence. Since TBEV was only found in the whole blood samples and not in the corresponding serum samples, TBEV most likely attaches to or infects some type of blood cell, and potentially remains there for at least 28 days in infected bank voles. A study by Krylova et al. [59] examined the interaction of different pathogenic strains with human blood samples in the first day after infection. A highly pathogenic strain of the TBEV-FE subtype showed rapid penetration and active reproduction in the blood cells, while a lowly pathogenic strain remained almost entirely in the serum fraction [59]. Thus, the interaction with the blood cells seems to contribute to the pathogenicity of TBEV. In addition to this, it is quite interesting that TBEV can remain in blood cells for a duration of 28 days despite the presence of neutralizing antibodies.

TBEV is known to rearrange intracellular cytoplasmic compartments in order to replicate in them, and these compartments are supposed to be inaccessible for the host immune system [60,61]. The antibodies circulating in the serum fraction of the blood might neutralize TBEV virions released from infected cells, but do not interfere with replication in the intracellular cytoplasmic compartments. Furthermore, the potential infection of naïve ticks is most likely not hindered by the presence of neutralizing antibodies [62], since co-feeding supposedly works through the transmission of infected cells [63]. One of the cell fractions infected during the co-feeding process is monocytes [63], and their interaction with TBEV has been well studied. They become infected with TBEV, show a multitude of structural changes in reaction to it [64], and can successfully transmit TBEV to laboratory mice [65]. Therefore, monocytes, the progenitor cells of macrophages, might be the location of replication of TBEV. However, since the findings of the present study were quite unexpected, the whole blood samples were frozen for RT-PCR testing and, therefore, the isolation of different cell fractions was not possible. Thus, the interaction of the virus with the host blood cells of the potential reservoir species bank vole should be part of future investigations. 

Four TBEV-Eu strains were simultaneously inoculated in two different evolutionary bank vole lineages to assess the influence of the vole origin when inoculated with virus strains isolated in areas where only one of both lineages naturally occurs. Some bank voles that were inoculated with the Battaune 17-H9 strain had to be euthanized prematurely, independently of the vole lineage. One of the voles died spontaneously, but did not display any neurological symptoms. Two additional bank voles of the Western lineage and one of the Carparthian lineage were euthanized within 12 days. Since one of the in-contact animals as well as one environmental control animal had to be taken out of the experiment prematurely, these early loses cannot be conclusively interpreted as being result of the TBEV infection, especially since the control animals tested negative by RT-PCR. However, the high viral RNA loads in nearly all organ samples of the inoculated bank voles strongly hinted at the involvement of TBEV in the death of one bank vole and the rapid weight loss of the other three inoculated animals. The reasons for the divergent behavior of this virus strain in comparison to the other strains used in the present study remain unknown, and additional animal experiments need to be performed to substantiate this phenomenon; however, the vole lineage did not appear to play a role. All of the bank voles of the Western as well as of the Carparthian lineage of the infection group that reached the endpoint of this study showed an RNAemia of at least 28 days. The virus strain Battaune 17-H9, which did not show any prominent amino acid substitutions in the envelope gene (data not shown) potentially leading to increased virus virulence, was isolated in Leipzig, Germany, where the Western vole linage is dominant [45,66]. Since bank voles of the Carpathian lineage showed a similar infection pattern, it seems that they are able to take on the role of their Western counterpart, which could be confirmed by using further strains. The strain CGl 223 was detected in the whole blood samples of some bank voles from both lineages, and the respective brain samples tested consistently negative. CGl 223 was isolated from the Slovak Republic, where the Carpathian vole lineage is primarily found [66]. Again, similar results for both lineages do not support an influence of different lineages on the TBEV transmission cycle. 

The strain IZ58, which was isolated from a region where TBEV is not considered to be endemic [47], led to no detection of viral RNA in either bank vole lineage at 28 dpi. A difference between the two lineages was only seen for the strain Scharl, which was originally isolated from the brain of a human. While all bank voles of the Western lineage remained negative in all samples, the brain as well as the spine samples of two of the bank voles of the Carpathian lineage were positive in the RT-PCR testing; however, clinical signs were not observed in any of the animals. Thus, the overall results of both vole lineages were quite similar for all simultaneously tested strains, which speaks against an influence of different lineages on the interaction between TBEV and its natural rodent host. With regard to virus transmission between the rodent hosts, it is highly unlikely that TBEV-Eu is transmitted horizontally, since none of the in-contact animals seroconverted, although the viral load seemed to be immense in the first week after infection and virus was successfully reisolated from selected organs. However, previous studies have described horizontal and vertical transmission between red voles when infected with a TBEV-Sib strain [58].

The animals that had to be euthanized early hinted at a systemic infection in the first week, with a neuroinvasion between days three and five. A week later, viral RNA was only detected in the whole blood samples, the brain/spine samples, and, surprisingly, the samples of the digestive tract. In line with that, TBEV has only recently been tentatively linked with gastrointestinal symptoms in humans [46]. Furthermore, humans can become infected with TBEV through the consumption of non-pasteurized dairy products [67], which indicates at least some degree of susceptibility of the gastrointestinal tract for TBEV infection. 

To relate the generated real-time PCR data to actual infectivity in cell culture, comparative analysis was performed. Overall, RT-PCR led to the detection of viral RNA in virus dilutions with a TCID_50_/mL as low as 10^−1.75^. This finding suggests that theoretically, even a single viral genome fragment could be detected with the presented RT-PCR. The organ samples collected from the animals that were taken out prior to the endpoint showed lower Cq values, leading to estimated TCID_50_/mL values that ranged from around 10^1.37^ to 10^2.92^. In accordance, virus reisolation on cell culture was successful. The viral genome that was detected 28 dpi, mainly in the brain samples, only correlated to TCID_50_/mL values of around 10^−0.18^ to 10^1.37^, complicating the reisolation in cell culture. Therefore, viral infectivity seems to decrease over the course of infection. However, Cq values of whole blood samples taken 28 dpi were comparable to the Cq values of whole blood samples from the animals that were taken out 5, 6, and 12 dpi, hinting at a consistent viremia throughout the course of 28 days. Cq values from the whole blood samples resulted from an extraction volume of 15 µL instead of the 100 µL that was used for organ samples and virus dilutions. Therefore, infectivity on cell culture may be even higher than estimated by this comparative analysis. To confirm this first estimation, additional experiments are needed in this now established animal model, investigating earlier time points in the course of infection of TBEV in bank voles.

In addition to the characterization of the virus–host interaction of different TBEV-Eu strains in the bank vole, the suitability of chest cavity lavage as a diagnostic material to detect neutralizing antibodies was investigated, since serum samples are not always available when animals die a natural death. Furthermore, such lavages are frequently used in epidemiological studies of wild caught animals when serum is not available [45,68]. The comparative testing of both sample matrices, i.e., serum and chest cavity lavage, showed that the chest cavity lavage does principally enable the detection of neutralizing antibodies. However, the values were far lower than the values that were detected in the serum samples of the same animal, which led to false negative results in seven bank voles. Therefore, the use of such lavage samples is convenient when no serum sample is available, but should be considered with caution for epidemiological studies due to its reduced sensitivity. For such studies, additional sample matrices should be validated to offer a reliable alternative to serum samples.

## 5. Conclusions

TBEV-Eu appears to be well adapted to the bank vole host, leading to long-lasting viremia and an infiltration of the brain without causing visible neurological symptoms. These findings fully support the role of bank voles as a reservoir host for TBEV, and encourage further research on this topic. 

## Figures and Tables

**Table 1:** Virus strains used in the present study, including information on the initial isolation (year, place, species).

Strain	First Isolation				Passage on Cell Culture	Accession Number (NCBI GenBank)	Reference
	Year	Country	Location	Species			
BaWa 15/943	2015	GER	Haselmühl	tick	1×	-	-
HB 171/11	2011	GER	Heselbach	tick	2×	KX268728	Dobler et al., 2016 [46]
IZ58	1965	GER	Schorfheide	tick	3×	-	Apitzsch et al., 1968 [47]
Neudörfl	1970	AUT	Neudörfl	tick	n.a.	U27495	Mandl et al., 1988 [48]
Battaune 17-H9	2017	GER	Leipzig	bank vole	1×	-	-
CGl 223	1990	SVK	Záhorská Ves	bank vole	1×	KC835597	Kozuch et al., 1995 [49]
HM 4-2	2015	GER	Haselmühl	bank vole	2×	-	-
Scharl	1956	AUT	Lower Austria	human	n.a.	-	Ecker et al., 1999 [12]
Langat virus	1956	MYS	Kuala Lumpur	tick	3×	-	Smith 1956 [43]

The number of passages on cell culture between first cultivation and the usage in the animal experiment is indicated. Passages of two isolates were not available (n.a.). The accession numbers refer to the full-length sequence of the respective strain. Austria: AUT, Germany: GER, Malaysia: MYS, SVK: Slovak Republic.

**Table 2:** Results of RT-PCR testing of all samples taken from inoculated bank voles at 28 days post infection. Groups with at least one positive sample are shaded in grey. S.int. and l. int.: small and large intestine.

Virus Strain	Bank Vole Lineage	Number of Positive Samples/Total Number															
		Blood	Serum	Brain	Spine	Lung	Heart	s. int.	l. int.	Liver	Spleen	Kidney	Bladder	Uterus	Faeces	Urine	Lavage
HM 4-2	Western	6/6	1/6	3/6	3/6	0/6	0/6	2/6	0/6	0/6	0/6	0/6	0/6	0/6 ^1^	0/6	0/6	0/6
BaWa 15/943	Western	1/6	0/6	1/6	0/6	0/6	0/6	0/6	0/6	0/6	0/6	0/6	0/6	0/6	0/6	0/4	0/6
Neudörfl	Western	4/6	0/6	0/6	0/6	0/6	0/6	0/6	0/6	0/6	0/6	0/6	0/6	0/6	0/6	0/2	0/6
HB 171/11	Western	6/6	0/6	1/6	0/6	1/6	0/6	0/6	0/6	0/6	0/6	1/6	0/6	0/6	0/6	0/5	0/6
Langat virus	Western	0/6	0/6	0/6	0/6	0/6	0/6	0/6	0/6	0/6	0/6	0/6	0/6	0/6 ^1^	0/5	0/1	0/6
Battaune 17-H9	Western	3/3	0/3	2/3	1/3	0/3	0/3	0/3	0/3	0/3	0/3	0/3	0/3	0/3	0/3	0/1	0/3
	Carparthian	5/5	0/5	2/5	2/5	0/5	2/5	1/5	2/5	0/5	1/5	0/5	0/5	0/5	0/4	0/1	0/5
CGl 223	Western	2/6	0/6	0/6	0/6	0/6	0/6	0/6	0/6	0/6	0/6	0/6	0/6	0/6	0/6	0/2	0/6
	Carparthian	4/6	1/6	0/6	0/6	0/6	0/6	0/6	0/6	0/6	1/6	0/6	0/6	0/6	0/5	0/1	1/6
IZ58	Western	0/6	0/6	0/6	0/6	0/6	0/6	0/6	0/6	0/6	0/6	0/6	0/6	0/6 ^1^	0/6	0/2	0/6
	Carparthian	0/6	0/6	0/6	0/6	0/6	0/6	0/6	0/6	0/6	0/6	0/6	0/6	0/6	0/6	0/0	0/6
Scharl	Western	0/6	0/6	0/6	0/6	0/6	0/6	0/6	0/6	0/6	0/6	0/6	0/6	0/6	0/6	0/2	0/6
	Carparthian	0/6	0/6	2/6	2/6	0/6	0/6	0/6	0/6	0/6	0/6	0/6	0/6	0/6	0/6	0/6	0/6
Σ	Σ	31/74	2/74	11/74	8/74	1/74	2/74	3/74	2/74	0/74	2/74	1/74	0/74	0/74	0/71	0/33	1/74

^1^ one animal was male; therefore, a testicle was sampled instead of the uterus.

**Table 3:** Results of the microneutralization assay comparing the usage of lavage samples to serum samples for the animals that had been inoculated with either the Neuddörfl, HM 4-2, HB171/11, or BaWa 15/943 TBEV-Eu strain or Langat virus. In-contact animals are shaded in grey. ND_50_: 50% neutralizing dose.

HM 4-2			BaWa 15/943			Neudörfl		
ID	serum	lavage	ID	serum	lavage	ID	serum	lavage
	ND_50_	ND_50_		ND_50_	ND_50_		ND_50_	ND_50_
2-1	1:960	1:5	24-1	1:480	1:10	1-1	1:1280	1:5
2-2	1:240	neg. ^1^	24-3	1:240	1:5	1-2	1:640	1:10
2-3	1:1280	1:7.5	24-4	1:320	1:20	1-3	1:240	1:5
2-4	neg. ^1^	neg. ^1^	24-2	neg. ^1^	neg. ^1^	1-4	neg.^1^	neg. ^1^
3-1	1:1280	1:40	25-1	1:80	1:5	6-1	1:960	1:10
3-2	1:1280	1:40	25-2	1:120	1:5	6-2	1:1280	1:7.5
3-3	1:1280	1:10	25-3	1:160	1:15	6-3	1:1280	1:30
3-4	neg. ^1^	neg. ^1^	25-4	neg. ^1^	neg. ^1^	6-4	neg. ^1^	neg. ^1^
HB 171/11			Langat virus					
ID	serum	lavage	ID	serum	lavage			
	ND_50_	ND_50_		ND_50_	ND_50_			
4-1	1:640	1:10	26-1	1:120	neg. ^1^			
4-2	1:1280	1:40	26-2	1:160	1:2.5			
4-3	1:640	1:5	26-3	1:240	1:2.5			
4-4	neg. ^1^	neg. ^1^	26-4	neg. ^1^	neg. ^1^			
5-1	1:160	1:2.5	27-1	1:160	neg. ^1^			
5-2	1:1280	1:5	27-3	1:320	1:10			
5-3	1:1280	1:40	27-4	1:320	1:7.5			
5-4	neg. ^1^	neg. ^1^	27-2	neg. ^1^	neg. ^1^			

^1^ neg. stands for a detection limit of <1:20 for serum samples and <1:5 for lavage samples.

**Table 4:** Results of the microneutralization assay comparing the usage of lavage samples to serum samples for the Battaune 17-H9, CGl 223, IZ58, and Scharl strains. The bank voles of the Western linage are compared to the bank voles of the Carpathian lineage. Contact animals are shaded in grey.

Battaune 17-H9						CGl 223					
Western lineage			Carpathian lineage			Western lineage			Carpathian lineage		
ID	serum	lavage	ID	serum	lavage	ID	serum	lavage	ID	serum	lavage
	ND_50_	ND_50_		ND_50_	ND_50_		ND_50_	ND_50_		ND_50_	ND_50_
15-1	1:40	1:10	8-1	1:160	1:2.5	17-1	1:60	1:2.5	10-1	1:30	1:7.5
15-2	n.a. ^2^	1:10	8-2	1:120	neg. ^1^	17-2	1:80	1:10	10-2	1:80	1:10
15-4	1:240	1:10	8-3	1:40	1:5	17-4	1:160	1:2.5	10-3	1:20	neg. ^1^
15-3	neg. ^1^	neg. ^1^	8-4	neg. ^1^	neg. ^1^	17-3	neg. ^1^	neg. ^1^	10-4	neg. ^1^	neg. ^1^
16-1	n.a. ^2^	1:20	9-2	1:80	1:10	18-2	1:160	1:7.5	11-1	1:40	1:5
16-2	n.a. ^2^	1:10	9-3	n.a. ^2^	1:15	18-3	1:160	1:5	11-2	1:160	1:5
16-3	1:160	1:15	9-4	1:240	1:2.5	18-4	1:160	1:5	11-4	1:320	neg. ^1^
16-4	neg. ^1^	neg. ^1^	9-1	neg. ^1^	neg. ^1^	18-1	neg. ^1^	neg. ^1^	11-3	neg. ^1^	neg. ^1^
IZ58						Scharl					
Western lineage			Carpathian lineage			Western lineage			Carpathian lineage		
ID	serum	lavage	ID	serum	lavage	ID	serum	lavage	ID	serum	lavage
	ND_50_	ND_50_		ND_50_	ND_50_		ND_50_	ND_50_		ND_50_	ND_50_
19-2	1:120	1:10	12-2	1:20	1:2.5	22-2	1:240	1:7.5	29-1	1:40	1:5
19-3	1:120	1:7.5	12-3	1:20	neg. ^1^	22-3	1:320	1:5	29-2	1:60	1:2.5
19-4	1:20	1:5	12-4	1:40	1:5	22-4	1:120	1:5	29-4	1:80	1:7.5
19-1	neg. ^1^	neg. ^1^	12-1	neg. ^1^	neg. ^1^	22-1	neg. ^1^	neg. ^1^	29-3	neg. ^1^	neg. ^1^
20-1	1:30	1:5	13-1	1:40	1:2.5	23-1	1:160	1:7.5	30-1	1:80	neg. ^1^
20-2	1:40	1:10	13-3	1:40	1:15	23-3	1:120	1.7.5	30-3	1:60	1:2.5
20-3	1:20	1: 5	13-4	1:80	neg. ^1^	23-4	1:320	1:5	30-4	1:20	neg. ^1^
20-4	neg. ^1^	neg. ^1^	13-2	neg. ^1^	neg. ^1^	23-2	neg. ^1^	neg. ^1^	30-2	neg. ^1^	neg. ^1^

^1^ neg. stands for a detection limit of <1:20 for serum samples and <1:5 for lavage samples. ^2^ Missing samples are marked with n.a. (not available).
